# The Predictive Creative Mind: A First Look at Spontaneous Predictions and Evaluations During Idea Generation

**DOI:** 10.3389/fpsyg.2019.02465

**Published:** 2019-11-01

**Authors:** Jacopo Valtulina, Alwin de Rooij

**Affiliations:** Department of Communication and Cognition, Tilburg School of Humanities and Digital Sciences, Tilburg University, Tilburg, Netherlands

**Keywords:** evaluation, idea generation, prediction, creativity, creative process, ideas

## Abstract

Idea generation, the process of creating and developing candidate solutions that when implemented can solve ill-defined and complex problems, plays a pivotal role in creativity and innovation. The algorithms that underlie classical evolutionary, cognitive, and process models of idea generation, however, appear too inefficient to effectively help solve the ill-defined and complex problems for which one would engage in idea generation. To address this, these classical models have recently been redesigned as forward models, drawing heavily on the “predictive mind” literature. These pose that more efficiency can be achieved by making predictions based on heuristics, previous experiences, and domain knowledge about what material to use to generate ideas with, and evaluate these subsequently generated ideas based on whether they indeed match the initial prediction. When a discrepancy occurs between prediction and evaluation, new predictions are made, and thus shaping what actions, and how these actions, are undertaken. Although promising, forward models of idea generation remain theoretical and thus no empirical evidence exists about whether such predictions and evaluations indeed form part of the idea generation process. To take a first empirical look at this, a mixed-methods study was conducted by analyzing people’s self-reports for the reasons of the actions that they take during an idea generation task. The results showed that predictions and evaluations are pervasive in the idea generation process. Specifically, switching between concept selection and conceptual combination and idea generation, as well as repeating idea generation based on earlier selected conceptual combination, and possibly (but to a lesser extent) concept selection and the repetition thereof, are likely to be driven by predictions and evaluations. Moreover, the frequencies of the predictions and evaluations that drive these actions influenced the amount of ideas generated, amount of concepts used, and within-concept fluency (the ratio of the amount of ideas generated per concept used). Therefore, the contribution of this paper is the first empirical evidence that indicates that the idea generation process is driven by both predictions and evaluations.

## Introduction

Idea generation, the process of creating and developing candidate solutions that when implemented can solve ill-defined and complex problems, plays a pivotal role in creativity and innovation (Isaksen et al., 2010; Mumford et al., 2012). Given its central role in creativity and innovation, it forms part of the skillset that enables people to develop the products that help the economy prosper, the medical interventions that help us live longer, and the artistic expressions that move us deeply (Abraham, 2018). There is no creativity and innovation without the generation of ideas (Isaksen et al., 2010; Mumford et al., 2012). It is therefore not surprising that research about creativity and innovation has always focused strongly on uncovering how idea generation works, and with that knowledge, how it can be best supported (Sawyer, 2011).

Classical evolutionary, cognitive, and creative process models that aim to explain the idea generation process, however, have recently been argued to have limited explanatory power (Gabora, 2011; Dietrich, 2015; Dietrich and Haider, 2015; Yang and Li, 2018). To address their limitations, a recent trend has been to redesign these classical models as forward models by explicitly including prediction and evaluation as part of the idea generation process (Abraham, 2018). In these models, prediction entails the pro-active anticipation of the consequences of a possible action for achieving a goal, whereas evaluation serves to assess whether the results of an action helped to achieve a goal as previously predicted (Dietrich, 2015; Dietrich and Haider, 2015). Discrepancies between prediction and evaluation, in turn, determine if and how an action is taken.

For example, when asked to develop original ideas for a viral online marketing campaign, one’s previous experiences may suggest that combining humorous content with shareable video are a recipe for success. When this prediction is confirmed, i.e., the ideas generated based on these concepts are evaluated as having a high potential for going viral, one is likely to continue this approach. However, when there is a negative discrepancy between this prediction and the evaluation of the generated ideas, i.e., combining humorous content with shareable video is evaluated to (no longer) lead to ideas that are likely to go viral, one is likely to change one’s approach and look for other concepts to work with.

Although this approach appears promising, it has remained purely theoretical until now. In the present paper, it is proposed that the manner in which the idea generation process is executed, which, according to creative process models, involves concept selection, conceptual combination, and the generation of ideas based on combined concepts (Mumford and McIntosh, 2017), is driven by prediction and evaluation. It is theorized that prediction and evaluation determine the degree of switching between concept selection and conceptual combination and idea generation, concept selection (and the repetition thereof), and conceptual combination and the generation of ideas (as well as the repetition thereof), which affects how ideas are generated (cf. Nijstad et al., 2010).

To provide empirical evidence for the developed conjectures, a first look is taken at whether and where predictions and evaluations are made during the idea generation process, and it is explored if and how these affect the way that the idea generation process is executed.

### From Blind Variation to Prediction–Action–Evaluation Cycles

Early models of the idea generation process were modeled after algorithms that also underlie biological evolution. Campbell (1960) proposed that creativity emerges from two alternating actions: (1) Blind variation, i.e., the undirected generation of variations on ideas (based on chance), and (2) selective retention, i.e., selecting generated ideas according to standards, of which new variations can be blindly generated in a subsequent iteration (BVSR model). A defining feature of the BVSR theory is that it assumes that selection imposes direction on variation, and that variation and selection are completely uncorrelated, i.e., not coupled in any way (Dietrich, 2015; Dietrich and Haider, 2015; for a thorough discussion, see Carruthers, 2018). The idea that variation is blind has been restated and adopted by many other scholars of creativity (see Simonton, 2011, for a review). However, recent work also suggests that models that propose that the generation of ideas and their evaluation are uncoupled are problematic (Gabora, 2011).

Dietrich and Haider (2015), for example, proposed that variation cannot be completely undirected and uncorrelated with selection. They argued that completely undirected variation would entail a too ineffective way to traverse the type of search space that characterizes the ill-defined and complex problems for which we engage in idea generation to solve. They therefore proposed to redesign the BVSR as a forward model based on the “predictive mind” literature (Hohwy, 2013; Clark, 2015). That is, if and how the actions that form part of the idea generation process are executed depends on ongoing predictions about what type of action is most likely to help achieve the best problem solution (Dietrich, 2015; Dietrich and Haider, 2015). Changing what and how an action is executed depends on the deviation of the evaluated outcomes of an action from what was previously predicted. Whether actions are invigorated, simply sustained, or abandoned depends on whether they are evaluated to be better, the same, or worse than previously predicted. Inclusion of prediction in variation can help explain how heuristics, previous experiences, and domain knowledge can play a role in effectively executing the idea generation process, as predictions made based thereupon can help traverse the type of search space needed to solve ill-defined and complex problems in a more efficient way.

Redesigning the BVSR as a forward model can explain this increased efficiency in the following way. First, generating variation is preceded by predictions that are based on heuristics, previous experiences, and domain knowledge that inform one about if, what, and how variation can help to best achieve the generation an appropriate idea. For example, one could favor making remote associations when producing variations to increase the likelihood that original ideas are generated, when the latter is one’s goal (Gibbert et al., 2012). This informs how variation is executed, i.e., though making remote associations. Second, the resulting variation that is produced is then evaluated on the basis of previously made predictions. For example, if making remote associations has led to the generation of original ideas, as predicted, one can continue with this way of producing variation. If it has led to more original ideas than expected, one may invigorate this approach. If this has not led to the predicted original ideas, a revision of the used strategy is needed, and thus the manner in which variation is executed needs to change, leading to renewed predictions and renewed execution of variation based on these predictions. This contrasts with blind variation because here, variation and selection are strongly coupled; heuristics, previous experiences, and domain knowledge are used to traverse through the search space in a more effective way than would be possible when people would generate variations blindly.

Complementarily, cognitive models of the idea generation process have been developed to explain how cognitive functions utilize knowledge as part of the generation of ideas (Abraham, 2018). Finke et al. (1992) proposed that ideas emerge via iteration through generative and exploratory phases under constraints imposed by knowledge about a product or problem solution that needs to be developed. In the generative phase, people generate various kinds of mental representations based on existing knowledge. The cognitive processes involved can include associative thinking (Mednick, 1962), retrieval (Perkins, 1994), and conceptual combination (Mobley et al., 1992). In the exploratory phase, people evaluate their ideas, which may include hypothesis testing and testing for limitations, and develop solutions from the evaluated mental representations. The exact thinking processes and actions used during different iterations depend on the state of development of a problem solution (Finke et al., 1992). For example, where early stages of the idea generation process may involve the generation of many basic representations through association, in later stages, evaluated associations can be recombined into new and more elaborate ideas (de Rooij and Vromans, 2018). As such, iteration through these two phases enables convergence upon creative ideas that help achieve set goals, by shifting the cognitive processes required to effectively execute each phase, and evaluating their success in terms of providing a solution within the given constraints.

Cognitive models such as those by Finke et al. (1992) were theoretically extended by Yang and Li (2018) and Wiggins and Bhattacharya (2014) to include prediction, to explain how constraints imposed by knowledge about a product or problem shape the idea generation process. That is, also classical cognitive models such as these have been recently redesigned as a forward model, and for the same reasons as the BVSR was redesigned. Specifically, it has been proposed that prediction, association, and selection shape the idea generation process (Wiggins and Bhattacharya, 2014; Yang and Li, 2018). The key assumption is that memory, and more broadly knowledge, serves prediction (Schacter et al., 2007). It has therefore been proposed that the key associations that underlie idea generation are learned during the idea generation process, and in turn enable convergence toward ideas that align with the goals and standards (e.g., standards of originality and effectiveness) of the person engaged in the idea generation process (Wiggins and Bhattacharya, 2014; Yang and Li, 2018). That is, predictions about the likelihood that associations between concepts enable the generation of ideas that align with the goals and standards of a person, inform what material is selected for idea generation. Subsequently, ideas are generated based on the selected material. This makes up the generative phase. The exploratory phase that follows consists of evaluating the generated ideas with regards to whether applied standards are met, and goals are likely to be achieved. In turn, the discrepancy between the initial predictions and later evaluations inform the value of the used associations in the idea generation process, and thus what material is selected in further iterations of the idea generation process. Indeed, when evaluation suggests that the generated idea is less likely to help achieve set goals than predicted, the associations used during idea generation will be less likely used in subsequent idea generation, whereas if associations were used to achieve a goal as predicted, they are more likely to be used in subsequent idea generation, and further built upon.

Process models of creativity define the actions that are commonly involved in creative problem solving, i.e., that lead to complete solutions that are both original and appropriate (Lubart, 2001). Consensus has been emerging about the actions that form part of the creative process (Lubart, 2001). Typically, these actions enable an individual to understand the problem that needs to be solved, generate ideas, and prepare for implementing one or more selected ideas; and evaluation is explicitly done after understanding the problem and after idea generation, to determine whether the problem is sufficiently understood and whether there are sufficiently suitable ideas, to continue with the next steps in the creative process (Isaksen et al., 2010). As such, the idea generation process forms part of the larger creative process (Mumford and McIntosh, 2017). The idea generation process, within these broader creative process models, is typically characterized by three actions: concept selection, concept combination, and idea generation (Mumford et al., 2012). Based on the knowledge obtained about a problem, people select concepts, which can include basic categories that associate with a problem, previous experiences, and cases related to the problem at hand. Selection matters, as the kinds of concepts that are later combined have implications for the originality and appropriateness of the ideas that are generated (Mumford et al., 1996; Gibbert et al., 2012). Selected concepts are then combined to create new knowledge (Abraham, 2018), on the basis of which ideas can be generated (Mumford et al., 1997). Note that idea generation, in the present paper, is therefore seen as only one action that forms part of a larger idea generation process (as is the case in evolutionary and cognitive models of the idea generation process).

The actions that form part of the creative process are not executed in a linear way (Mumford and McIntosh, 2017). Rather, people move back and forth between the actions taken, often for reasons yet unknown (Mumford et al., 2012). Interestingly, forecasting, the deliberate anticipation of potential consequences of the implementation of an idea, is studied as a technique aimed to support the execution of the creative process (McIntosh et al., 2019; Todd et al., 2019). Specifically, forecasting has been studied as a technique to support idea evaluation, where forecasting the consequences of implementing ideas is used to gather information in order to make decisions about what ideas can be implemented, should be revised, or not pursued at all (Bruijninckx and de Rooij, unpublished). Note that, indeed, the definitions of forecasting and prediction as used in the present paper overlap, with the distinction that prediction, as used in the present paper, is not necessarily deliberate or used as a creative technique, but rather as a key psychological process that is needed to generate ideas. However, these same creative process models also propose that one moves forward and backward in non-linear ways between the actions that form part of the idea generation process (Mumford et al., 2012). Surely, there must also be reasons for why concepts are selected or not for subsequent conceptual combination and idea generation, and why ideas are being generated based on the same conceptual combinations or not. Indeed, executing the steps in the idea generation process in a non-linear fashion likely requires that prediction and evaluation are a pervasive part of this process – for the same reasons that evolutionary (Dietrich, 2015, 2019; Dietrich and Haider, 2015) and cognitive models of idea generation (Wiggins and Bhattacharya, 2014; Yang and Li, 2018) require prediction to couple generation to evaluation.

As such, it is clear that the prediction and evaluation are likely a necessary component of the idea generation process, and more recent theoretical models of the idea generation process acknowledge this. Empirical evidence for these conjectures, however, is still lacking.

### The Present Study

In the present study, a first look is therefore taken at whether and where spontaneous predictions and evaluations may drive changes in the actions that form part of the idea generation process, and if and how these affect the way that the idea generation process is executed. It is proposed that how people cycle through the actions concept selection and conceptual combination and idea generation shapes the idea generation process through prediction and evaluation in at least three ways:

(1) Switching between concept selection and conceptual combination and idea generation.
(2) Concept selection (and the repetition thereof).
(3) Conceptual combination and idea generation (and the repetition thereof).

Firstly, during concept selection, predictions are made about the likelihood that a concept (in combination with other concepts) will facilitate conceptual combination in a manner that enables effective idea generation (Isaksen et al., 2010) or the emergence of ideas that solve a given problem (Mumford et al., 1996). If this likelihood appears high, the concept is selected for conceptual combination, and one or more ideas are generated based thereupon. That is, one switches from concept selection to conceptual combination and idea generation. Ideas that are generated (or the lack thereof) are subsequently evaluated. When the selected concepts do not (any more) facilitate the generation of ideas in a manner as previously predicted, i.e., there is a discrepancy between the predictions made about the selected concept(s) and the evaluation of ideas that can be generated based thereupon, one switches back from conceptual combination and idea generation to concept selection to retrieve different concepts to continue the idea generation process. As such, it is conjectured that prediction and evaluation drive switching between the actions concept selection and conceptual combination and idea generation.

Secondly, during concept selection, predictions are made on the basis of certain heuristics, previous experiences, and domain knowledge. When it is assumed that concepts should facilitate the execution of such heuristics (e.g., selecting remote associations), or need to match previous experiences (e.g., similar concepts have not worked well during the present idea generation process), and domain knowledge (e.g., previous experiences indicate that a concept is commonly found in failed solutions) about what is likely to solve a given problem (Mumford et al., 1996; Gibbert et al., 2012), selecting or not selecting a concept can also be based on evaluations of whether predictions about the efficacy of one’s heuristics, previous experiences, and domain knowledge match with a concept that is retrieved or not. When this is not the case, we expect to observe the action that people continue their search for suitable concepts, i.e., execute the action of concept selection (and the repetition thereof).

Thirdly, during the generation of ideas, predictions that are made about the likelihood that a conceptual combination enables the (continued) generation of ideas (that are likely to help solve a given problem) can be updated based on evaluations of whether the ideas that are generated still match earlier predictions. As long as this is the case (they match), idea generation based on the same conceptual combination continues; i.e., one executes the actions conceptual combination and idea generation (and the repetition thereof).

If the above conjectures truly form part of the idea generation process, one should be able to frequently find these predictions and evaluation in the reasons that people report about the actions that they take as part of the idea generation process, and affect the way in which the idea generation process is executed as detailed in the above.

## Method

To take a first look at spontaneous predictions and evaluations during the idea generation process, the reasons that underlie taking actions during this process were explored in a mixed-method study.

### Participants

Sixty^1^ people initially participated in the study. However, the data of three participants were not used in further analysis because these participants did not follow the instructions provided. This resulted in a sample of 57 people (*M*_*age*_ = 22.78, *SD*_*age*_ = 2.65, 16 males, 41 females). The participants were recruited via the participant recruitment system of the Communication and Information Science program at Tilburg University. As a consequence, all participants were students of the Communication and Information Science program and thus engaged in higher education. The participants received study credits in exchange for their participation. Given that creativity depends on domain specific knowledge (Rietzschel et al., 2007), and the creative task that the participants will engage with is marketing task, the participants were asked to self-report their marketing experience. The participant’s self-reported expertise in marketing was moderate 2.65 (*SD* = 0.96). The study was approved by the Research Ethics and Data Management Committee of the Tilburg School of Humanities and Digital Sciences, Tilburg University.

### Materials and Measurements

#### Creative Task

A novel creative task was developed to enable participants to self-report the reasons for their actions during the idea generation process. This creative task consisted of four activities: (1) reading a written briefing about a project for which participants would generate ideas, (2) an association task, (3) the actual idea generation task, and (4) an idea selection task.

##### Briefing

**FIGURE 1:**
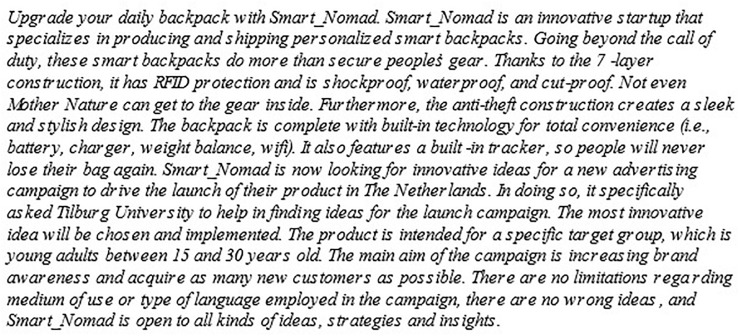
Briefing about the creative problem that needed to be solved.

The creative task started with the instruction that the participants would help to generate ideas for an advertising campaign to promote a product for the (fictional) business Smart_Nomad. This product was a “personalized smart backpack.” This topic was chosen because the participants to which there was access to for this study all have some degree of experience in marketing, advertising, and branding due to the curriculum of the study program they are participating in. The instructions were followed by a briefing supposedly provided by Smart_Nomad. The briefing is presented in Figure 1.

##### Association task

Before generating ideas, participants were asked to write down as many associations they could come up with based on the information provided in the briefing in 10 min. The associations could range from basic categories, personal experiences, and elements from prior cases (e.g., in marketing or branding). We hitherto refer to these as concepts. These concepts would later be used in the idea generation task, by asking participants to select these and combine these associations into new knowledge based on which they could generate ideas (Abraham, 2018). Participants were asked to report to the researcher when they had difficulty listing (more) concepts, at which time the researcher would provide creative primes to facilitate the continued listing of associations. The primes were funny, serious, case-related, or case-unrelated and were written on a sheet of paper that the participants could choose from (e.g., millennials, prank, laptop, fire, picnic) (Brown and Bhadury, 2006). Maximizing the amount of concepts listed helped to ensure that there was enough material for the participants to combine during the subsequent idea generation task. Each concept was written down on a white memo sheet.

##### Idea generation task

**FIGURE 2:**
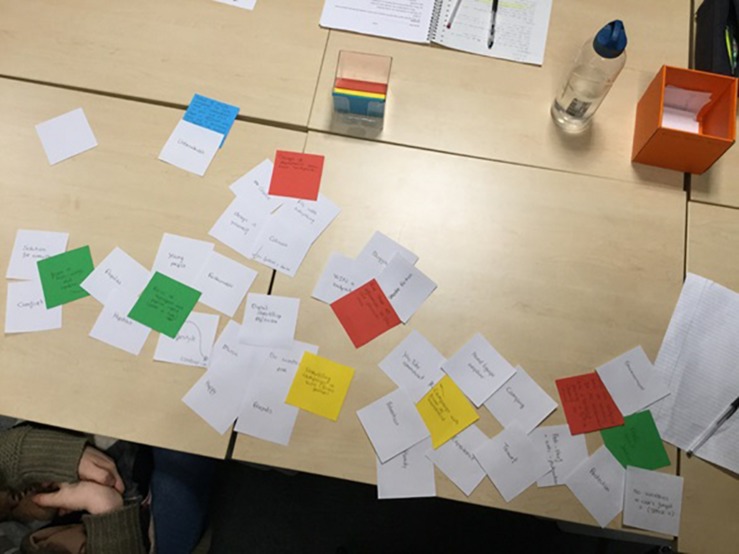
Table setup developed for use during the creative task.

After the association task, the participants engaged in an idea generation task. The goal of this task was to write down as many creative, innovative, original, and usable solutions based on the briefing by Smart_Nomad as possible in 15 min. As part of their idea generation process, the participants were instructed to select concepts that they wrote down during the association task, and combine these concepts to form new knowledge based on which to generate ideas for the problem provided in the briefing. When an idea was generated, it was written down on a red, yellow, or green memo sheet and positioned on the table. Participants were asked to place the concepts they combined into an idea with each idea on the table. Newly generated concepts that were used during the actions conceptual combination and idea generation were written down on blue memo sheets and also placed next to the ideas they were used for. This way, participants externalized their idea generation process in a manner that approaches the way people think about how concepts and ideas relate (cf. Dove et al., 2018). This was developed through iterative pilot testing as participants initially, without this way of externalizing their idea generation process, were not sufficiently able to indicate the reasons that underlie taking actions during their idea generation process. A visual impression of the setup and use of memo sheets is presented in Figure 2.

##### Idea selection task

After the idea generation task, participants were asked to evaluate their ideas and select, based on the briefing, the one idea that they would recommend for implementation to Smart_Nomad. This served no purpose other than to improve the construct and ecological validity of the task, as otherwise there would not be a final goal and motivator for generating ideas, which is typical for the idea generation process, because it is normally embedded in a larger creative process, and followed by an explicit idea evaluation and selection activities, and later implementation.

#### Probing Prediction and Evaluation

**FIGURE 3:**
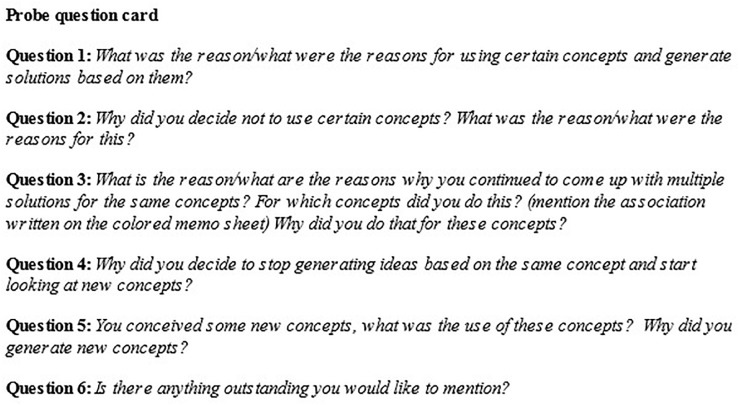
Probe questions used by the researcher to interrogate the reasons for actions taken during the idea generation task.

To capture the reasons that underlie taking actions during the idea generation process that could be indicative of the presence of spontaneous predictions and evaluations therein, the idea generation task was briefly stopped at three moments. During these moments, the participants were asked to tell the researcher about the reasons for taking specific actions during their idea generation process in the time prior to each probe. That is, between the start of the task and probe 1, between probe 1 and probe 2, and between probe 2 and probe 3. Questions were not explicitly formulated to address the prediction and evaluation in order to avoid potential bias in the answers. Rather, each question asked about the reason for a particular action done as part of the idea generation process. In particular, participants were asked about the reasons for (not) using certain concepts for conceptual combination, and about stopping or continuing idea generation based on the same conceptual combination. Moreover, they were asked about the conception of new concepts and whether there was further information they wanted to provide. Pilot testing showed that participants disclosed more about their actions if they were verbally asked to answer the questions instead of asking them to write down an answer. For this reason, the questions were administered verbally and transcribed by the researcher. The questions are presented in Figure 3.

The probes occurred at random moments within the following time slots: minutes 3–5, minutes 8–10, and minutes 13–15. After the first probe, participants switched from writing down their idea on red to yellow memo sheets. After the second probe, participants switched from writing down their ideas on yellow to green memo sheets. The use of these colored sticky notes was not counterbalanced. Directly after the third probe, the idea generation task was stopped. As part of each probe, the researcher took a picture of the table on which the ideas and concepts were displayed, as to enable further analysis of how the idea generation process was executed.

#### Capturing Idea Generation Process Execution

To capture how the idea generation process was executed and assess how this was affected by prediction and evaluation, three quantitative performance metrics were coded for the output that was generated prior to each probe: fluency, flexibility, and within-concept fluency. Fluency was assessed by counting the number of ideas generated by each participant (Guilford, 1967), which, in the present study, simply meant counting the red, yellow, and green memo sheets. This indicates the ease with which participants were able to generate ideas, which is commonly assumed to increase the chance that creative ideas are developed (Plucker et al., 2011). Flexibility was assessed by counting the number of different concepts used when producing ideas by each participant (Guilford, 1967), which, in the present study, simply meant counting the amount of white memo sheets placed with the red, yellow, and green sheets. This indicates a strategy commonly applied during the idea generation process where the inclusion of many different concepts increases the chance of generating a creative idea (Nijstad et al., 2010). Within-concept fluency was calculated by dividing fluency by flexibility for each participant. This was used to indicate another strategy commonly applied during the idea generation process, where multiple ideas are typically generated by exploiting only a few (successful) concepts (Nijstad et al., 2010).

#### Socio-Demographics

To gain insight into the characteristics of the sample, basic socio-demographics were captured with a questionnaire prior to the study. Specifically, participants were asked to write down their age and gender. The participants’ experience in marketing was measured on four 5-point Likert scales (1 = very inexperienced, 5 = very experienced). Each inquires about the participants’ experience in professional marketing, advertising, social media marketing, and branding. Cronbach alpha suggested that the internal consistency was good, α = 0.88.

### Procedure

Upon arrival, participants were seated at a table. They were provided a written information sheet about the study and their rights related to their participation and data handling, signed informed consent, and filled in a brief questionnaire about their socio-demographics. At this stage, information that could reveal the true purpose of the study was withheld. Instructions about how the creative task should be executed were provided, after which participants engaged in the creative task. First, they received the briefing and took ample time to read and understand it. Second, instructions were briefly repeated on how to execute the association task, and the participants executed the association task. Third, instructions were briefly repeated on how to execute the idea generation task, after which the participants executed the idea generation task, and during which time the three probes were administered. A researcher was present throughout the task in case there were questions, or participants needed help with something, and to administer the probes. After administering the third probe, the idea generation task ended. Fourth, the participants selected one of their ideas to be recommended for implementation by Smart_Nomad. After the creative task ended, the participants were debriefed about the aspects of the study that included deception and its overall purpose. In total, participation took approximately 45 min.

### Analysis

The self-reported reasons that underlie the actions taken during the idea generation process were coded into variables indicative of whether a given reason indicated if a prediction happened or not, and whether it indicated if an evaluation happened or not. The definitions of “prediction” and “evaluation” were obtained through the grammatical interpretation of the participant’s responses. On one hand, whenever a participant used the future tense or modal auxiliary verbs to describe the use of a particular concept (e.g., would), then a prediction happened (cf. Gotti, 2006; Kakzhanova, 2013). This is due to the fact that it is used to talk about the future in the past and to express the conditional mood. On the other hand, whenever a participant used the past or the present tense to describe concepts, ideas, or their use, an evaluation happened (cf. Howe, 1966). When both occurred in the same response, the grammar features related to “evaluation” outweighed the “prediction” features; therefore, the response was considered an “evaluation.” To assess the intra-observer agreement, a random selection of 15% of the responses was recoded 2 weeks after the first coding. Cohen’s kappa suggested substantial intra-observer agreement for predictions found in answers to question 1, κ = 0.71, but moderate agreement for evaluations found in answers to question 4, κ = 0.43, and substantial agreement for predictions found in answers to question 2, κ = 0.76, and evaluations found in answers to question 2, κ = 0.66, and for predictions found in answers to question 3, κ = 0.77, and evaluations found in answers to question 3, κ = 0.78. Answers to questions 5 and 6 were not used in the analysis.

To explore how prediction and evaluation correlated with the way in which the idea generation process was executed, generalized linear mixed modeling was used. Full factorial models were calculated for the coded predictions and evaluations that were hypothesized to relate to individual actions: Predictions (question 1) and evaluations (question 4) conjectured to drive switching between the actions concept selection and conceptual combination and idea generation, predictions and evaluations (question 2) conjectured to drive concept selection (and the repetition thereof), and predictions and evaluations (question 3) conjectured to drive the actions conceptual combination and idea generation (and the repetition thereof). These were entered as dummy-coded nominal variables (prediction: yes = 1, no = 0; evaluation: yes = 1, no = 0). The intercepts were modeled as random effects. Degrees of freedom were fixed for all tests (residual method). The number of the probe (1–3) was entered as the repeated measures variable. Note, however, that repeated measures were not analyzed to compare differences between the different probes, but rather for appropriate aggregation. For each full factorial model, results were calculated for the dependent variables fluency, flexibility, and within-concept fluency. The covariance matrix (diagonal) was selected by minimizing the Akaike information criterion and accepting a more complicated covariance matrix only when the additional degrees of freedom used yielded a significant decrease in the information criterion number, following the procedure outlined in Field (2013).

## Results

Predictions and evaluations were frequently reported in the reasons for switching between the concept selection and conceptual combination and idea generation. That is, predictions about what concepts would facilitate creative idea generation were reported together with evaluations that concept combinations do not support creative idea generation (any more) in 48% of the probes (Table 1, left). In contrast, 16% (*n* = 28) only reported these predictions, 22% (*n* = 37) reported only these evaluations, and 14% reported neither in the probes. These findings suggest that predictions about what concepts facilitate idea generation, and relatedly evaluations of the utility of combinations concepts for idea evaluation commonly form part of the idea generation process. Example quotes are presented in Table 2, component 1.

**TABLE 1:** Frequencies of predictions and evaluations that were conjectured to drive switching between the actions concept selection and conceptual combination and idea generation (action 1), concept selection (and the repetition thereof) (action 2), and conceptual combination and idea generation (and the repetition thereof) (action 3).

		Evaluation		
		
		No	Yes	Total
**Action 1**				
Prediction	No	24	37	61
	Yes	28	82	110
Total		52	119	171
**Action 2**				
Prediction	No	24	120	144
	Yes	2	25	27
Total		26	145	171
**Action 3**				
Prediction	No	31	41	72
	Yes	42	57	99
Total		73	98	171

**Frequencies are the counts of the responses each of the three probes of the 57 participants.**

**TABLE 2:** Example quotes that illustrate the type of predictions and evaluations made during the idea generation process.

**Type**	**Example quote**
**Action 1: Switching between concept selection and conceptual combination and idea generation**	
Prediction	*“I thought about targeting the right customer, I think creating an online community as a form of organic marketing will increase positive word-of-mouth.”*
	*“Instagram is, like, a bigger thing, it is more useful, deep, it will make it easier to have new ideas.”*
Evaluation	*“At first I thought it could work, but now that I look at it, I think that using ads in specific magazines doesn’t convince me anymore.”*
	“*When I came up with it, I thought it could still come in handy, but now I think I won’t get new possibilities of use.”*
**Action 2: Concept selection (and the repetition thereof)**	
Prediction	*“Previously yes, but then I thought that Gamers would not fit in the ultimate product target group.*”
	*“I just know it will not help to have better insight on the product.”*
Evaluation	*“Fashionable doesn’t address the right audience.”*
	*“Gaming; Awareness in other countries, these are not prime goals, I can’t use them.”*
**Action 3: Concept combination and idea generation (and the repetition thereof)**	
Prediction	*“Wi-Fi and scientific improvement together will be meaningful features when communicating the product to people.”*
	*“I clustered them, I thought they would make more sense if used together. The same for the other clusters for the other ideas.”*
Evaluation	*“Marketing is an enormous concept, it connects with many others, that is why it was possible to get more ideas for the campaign.”*
	*“I think these concepts are important if taken singularly, better to focus on once concept, stress it, and then use another one to come up with strategies.”*

Prediction, but evaluations in particular were reported in the reasons for concept selection (and the repetition thereof). That is, predictions about what concepts are unlikely to facilitate the idea generation process were reported together with evaluations that concepts should not be selected for concept combination and subsequent idea generation in only 15% (*n* = 25) of these probes (Table 1, middle). Less than 1% (*n* = 2) of the probes were coded as predictions only, and 14% (*n* = 24) as neither. In contrast, evaluations indicating reasons for not selecting concepts were reported in 70% (*n* = 120) of the probes. This suggests that not selecting concepts was mostly driven by evaluations, at least as indicated by the data obtained in the present study, although predictions, albeit less commonly, also form part of not selecting concepts for idea generation. Example quotes are presented in Table 2, component 2.

Furthermore, predictions and evaluations were frequently reported in the reasons for combining concepts and generating ideas based thereupon (and the repetition thereof). That is, predictions about the continued use of the same concepts for idea generation were reported together with evaluations suggesting that the same concepts can facilitate (continued) idea generation in 33% (*n* = 57) of the probes (Table 1, right). In contrast, 25% (*n* = 42) of the probes suggested that only these predictions occurred, whereas in 24% (*n* = 41), only these evaluations were reported, and in 18% (*n* = 31), neither were reported. This suggests that predictions about the continued use of selected concepts for idea generation, and evaluations about whether these concepts can be used to facilitate (continued) idea generation, also commonly form part of the idea generation process. Example quotes are presented in Table 2, component 3.

The predictions and evaluations that underlie the actions that form part of the idea generation process also affect the manner in which the idea generation process is executed. The descriptive statistics are presented in Tables 3, 4.

**TABLE 3:** Means and standard errors for fluency, flexibility, and within concept fluency for predictions, evaluations, and their interaction.

		**Fluency**		**Flexibility**		**Within-concept fluency**	
		***M***	***SD***	***M***	***SD***	***M***	***SD***
**Action 1: Switching between concept selection and conceptual combination and idea generation**							
Prediction	No	8.10	3.28	20.92	5.33	0.40	0.18
	Yes	8.25	3.06	20.55	5.61	0.41	0.15
Evaluation	No	8.27	3.45	21.52	6.02	0.40	0.16
	Yes	8.16	2.99	20.32	5.24	0.41	0.16
**Action 2: Concept selection (and the repetition thereof)**							
Prediction	No	8.15	3.12	20.69	5.38	0.40	0.15
	Yes	8.41	3.25	20.63	6.22	0.43	0.19
Evaluation	No	8.92	3.48	20.08	5.36	0.45	0.16
	Yes	8.06	3.06	20.79	5.53	0.40	0.16
**Action 3: Concept combination and idea generation (and the repetition thereof)**							
Prediction	No	8.50	3.38	21.33	5.97	0.41	0.17
	Yes	7.97	2.93	20.21	5.11	0.40	0.15
Evaluation	No	7.79	2.81	20.89	5.05	0.38	0.13
	Yes	8.49	3.33	20.53	5.83	0.43	0.17

**TABLE 4:** Pearson correlations (two-tailed) between fluency, flexibility, and within-concept fluency.

	**Fluency**	**Flexibility**	**Within-concept fluency**
Fluency	–		
Flexibility	0.326^∗^	–	
Within-concept fluency	0.804^∗∗^	−0.270^∗^	–

**Note that these variables were aggregated (sum) across the three measurement times (probes). ^∗^p < 0.05, ^∗∗^p < 0.01.**

*Model 1* tested the effects of predictions about what concepts would facilitate idea generation, and evaluations of whether concept combinations did not support idea generation, on fluency, flexibility, and within-concept fluency (Table 5, model 1).

**TABLE 5:** Results of the generalized linear mixed model analysis for the correlations between predictions and evaluations with fluency, flexibility, and within-concept fluency.

	**Fluency**	**Flexibility**	**Within-concept fluency**
**Model 1: Switching between concept selection and conceptual combination and idea generation**			
Intercept	7.86^∗∗^(0.34)	20.05^∗∗^(0.60)	0.40^∗∗^(0.02)
Predictions	0.94 (0.61)	0.83 (1.10)	0.04 (0.03)
Evaluations	1.47^∗^ (0.67)	1.93 (1.20)	0.04 (0.03)
Predictions x Evaluations	−3.19^∗∗^ (1.05)	−1.73(1.89)	−0.14^∗^ (0.05)
**Model 2: Concept selection (and the repetition thereof)**			
Intercept	8.47^∗∗^(.63)	20.87^∗∗^(1.11)	0.43^∗∗^(0.03)
Predictions	−0.50(0.69)	−0.09(1.22)	−0.04(0.04)
Evaluations	−0.98(2.31)	−3.40(4.06)	−0.01(0.12)
Predictions x Evaluations	2.05 (2.41)	2.90 (4.24)	0.06 (0.12)
**Model 3: Concept combination and idea generation (and the repetition thereof)**			
Intercept	8.02^∗∗^(0.41)	20.66^∗∗^(0.72)	0.40^∗∗^(0.02)
Predictions	1.13^†^ (0.64)	−0.31(1.12)	0.06^†^ (0.03)
Evaluations	−0.12(0.63)	−1.07(1.11)	0.01 (0.03)
Predictions x Evaluations	−1.38(0.97)	3.78^∗^ (1.71)	−0.12^∗^ (0.05)

**^†^p < 0.10, ^∗^ p < 0.05, ^∗∗^ p < 0.01.**

The results showed that the occurrence of the evaluations significantly and positively correlated with fluency, *B* = 1.47, *t*(167) = 2.19, *p* = 0.030.

Moreover, the results showed a significant interaction effect on fluency, *B* = −3.19, *t*(167) = 3.03, *p* = 0.003. Pairwise comparisons (sequential Sidak) showed that when no predictions and no evaluations were reported (*M* = 7.08, *SE* = 0.63), people had generated less ideas than when no predictions, but evaluations were reported (*M* = 8.80, *SE* = 0.51), *t*(167) = 2.12, *p* = 0.036; and people had generated less ideas than when predictions, but no evaluations were reported (*M* = 9.33, *SE* = 0.58), *t*(167) = 2.63, *p* = 0.009. Moreover, when both predictions and evaluations were reported (*M* = 7.86, *SE* = 0.34), participants had generated less ideas than when predictions but not evaluations were reported (*M* = 9.33, *SE* = 0.58), *t*(167) = 2.19, *p* = 0.030, and when no predictions but only evaluations were reported (*M* = 8.80, *SE* = 0.51), albeit not significantly in the latter, *t*(167) = 1.54, *p* = 0.126.

The results also showed a significant interaction effect on within-concept fluency, *B* = −0.14, *t*(167) = 2.52, *p* = 0.013. Pairwise comparisons (sequential Sidak) showed that when no predictions and no evaluations were reported (*M* = 0.34, *SE* = 0.03), participants had generated less ideas per concept than when they reported no predictions but only evaluations (*M* = 0.44, *SE* = 0.03), *t*(167) = 2.31, *p* = 0.022, and when they reported predictions but no evaluations (*M* = 0.44, *SE* = 0.03), *t*(167) = 2.45, *p* = 0.026. Moreover, no significant difference was found between when both predictions and evaluations were reported (*M* = 0.40, *SE* = 0.02), with when predictions but no evaluations were reported (*M* = 0.44, *SE* = 0.03), *t*(167) = 1.16, *p* = 0.249, and with when no predictions but only evaluations were reported (*M* = 0.44, *SE* = 0.03), *t*(167) = 1.19, *p* = 0.237.

*Model 2* tested the effects of predictions about what concepts are unlikely to facilitate creative idea generation, and evaluations of whether concepts should not be selected for concept combination and subsequent idea generation, on fluency, flexibility, and within-concept fluency. The results showed no significant effects of these predictions, evaluations, or their interaction on fluency, flexibility, and within-concept fluency (Table 5, model 2).

*Model 3* tested the effects of predictions about the continued use of the same concepts for idea generation, and evaluations suggesting that the same concepts can facilitate (continued) idea generation, on fluency, flexibility, and within-concept fluency (Table 5, model 3).

The results showed no significant correlations of predictions or evaluations with fluency, flexibility, and within-concept fluency. However, a non-significant positive correlation between predictions and fluency, *B* = 1.13, *t*(167) = 1.78, *p* = 0.077, and a non-significant positive correlation between predictions and within-concept fluency, *B* = 0.06, *t*(167) = 1.88, *p* = 0.062, may be of interest.

The results, however, did show a significant interaction effect on flexibility, *B* = 3.38, *t*(167) = 1.98, *p* = 0.049. Pairwise comparisons (sequential Sidak) showed that when no predictions and no evaluations were reported, people had used more concepts during idea generation (*M* = 22.65, *SE* = 0.98) than when no predictions but only evaluations were reported (*M* = 20.35, *SE* = 0.85), *t*(167) = 1.78, *p* = 0.077, and when predictions but no evaluations were reported (*M* = 19.59, *SE* = 0.84), *t*(167) = 2.38, *p* = 0.019, albeit not significantly in the former. Moreover, when both predictions and evaluations were reported, participants had used more concepts (*M* = 20.66, *SE* = 0.72) than when predictions but no evaluations were reported (*M* = 19.59, *SE* = 0.84), *t*(167) = 0.97, *p* = 0.335, and when no predictions but only evaluations were reported (*M* = 20.35, *SE* = 0.85), *t*(167) = 0.28, *p* = 0.780. These were, however, not significant.

Because flexibility positively correlates with fluency (Table 4), it was also explored whether the found effects of predictions about the continued use of the same concepts for idea generation, and evaluations suggesting that the same concepts can facilitate (continued) idea generation, on flexibility were not driven by fluency. This is to help rule out alternative explanations. This was done by adding fluency as a covariate to *model 3*. The results again showed no significant coefficients for predictions, *B* = −1.01, *t*(166) = −0.96, *p* = 0.340, or evaluations, *B* = −1.01, *t*(166) = −0.97, *p* = 0.335. However, in this model, the correlation between fluency and flexibility was replicated, *B* = 0.61, *t*(166) = 4.79, *p* < 0.001. Moreover, the results replicated the interaction effect, *B* = 4.23, *t*(166) = 2.62, *p* = 0.010. These findings suggest that the found effects of prediction and evaluation on flexibility are not only driven by fluency.

Complementarily, the results showed a significant interaction effect on within-concept fluency, *B* = −0.12, *t*(167) = 2.53, *p* = 0.013. Pairwise comparisons (sequential Sidak) showed that when no predictions and no evaluations were reported (*M* = 0.35, *SE* = 0.03), participants had generated less ideas per concept than when no predictions but only evaluations were reported (*M* = 0.46, *SE* = 0.02), *t*(167) = 3.17, *p* = 0.002, and when predictions but no evaluations were reported (*M* = 0.41, *SE* = 0.02), *t*(167) = 1.72, *p* = 0.088, albeit not significantly in the latter. However, when both predictions and evaluations were reported (*M* = 0.40, *SE* = 0.02), participants generated a similar amount of ideas per concept when prediction but no evaluation was reported (*M* = 0.41, *SE* = 0.02), *t*(167) = 0.18, *p* = 0.861, and generated less ideas per concept when no predictions but only evaluations were reported (*M* = 0.46, *SE* = 0.02), *t*(167) = 1.88, *p* = 0.062. However, these were also not significant.

## Discussion

The presented study aimed to take a first look at whether and where predictions and evaluations are made about the actions taken during the idea generation process, and if and how these affect the way that the idea generation process is executed.

### Summary and Interpretation of the Results

The results showed overall that predictions and evaluations happen spontaneously and frequently during the idea generation process. The results furthermore confirm our conjectures that different patterns of predictions and evaluations exist within the idea generation process and underlie a specific set of actions.

First, the results indicated that switching between the actions concept selection and conceptual combination and idea generation is driven by both predictions and evaluations. That is, predictions about what concepts would facilitate idea generation and evaluations of whether concept combinations did not support idea generation (any more) occurred frequently in the participant’s reports about the reasons for their actions taken during the idea generation process. These predictions and evaluations also correlated with how the idea generation process was executed. Participants that reported evaluations more frequently also generated more ideas. When participants reported no evaluations and no predictions, they had also generated less ideas than when they reported only predictions or only evaluations. Moreover, when both predictions and evaluations were reported, they had generated less ideas than when predictions but not evaluations were reported. No correlations were found of these predictions and evaluations with the amount of concepts used. Complementarily, when participants reported no evaluations and no predictions, they had generated less ideas per selected concept than when they reported only predictions or only evaluations. However, no differences were found for the amount of ideas generated per concept between participants that reported both predictions and evaluations, with reporting predictions or reporting evaluations only.

Speculatively, these findings can be explained in the following ways. The found relationship between a lack of predictions and evaluations and the generation of less ideas and lowered within-concept fluency may simply suggest that the idea generation process is stagnating. Whereas being actively engaged in the idea generation process involves predictions and evaluations. This conjecture is supported further by a positive correlation between evaluations and the amount of generated ideas. Interestingly, the finding that when participants reported both predictions and evaluations, they had generated less ideas than when only predictions but no evaluations were reported, suggests that frequent switching may be the consequence of a problematically executed idea generation process. That is, when prediction in one probe did not coincide with an evaluation, this may indicate continued idea generation thereafter and, vice versa, may indicate that continued idea generation preceded sustained idea generation. Thus, when both prediction and evaluation happen as frequently as to report them in the same probe, this may signal issues during the execution of the idea generation process. This is in line with previous research that suggests that the frequency of switching between concept selection and subsequent conceptual combination and idea generation can affect the generation of ideas (e.g., Nusbaum and Silvia, 2011; Beaty et al., 2014). The findings of the present study add that switching can be driven explicitly by spontaneous predictions about the likelihood that a concept will facilitate the generation of ideas and evaluations that a conceptual combination will not facilitate creative idea generation (any more).

Second, the results obtained in the present study indicated that the action of concept selection (and the repetition thereof) can be driven by predictions and evaluations in particular. That is, predictions about what concepts are unlikely to facilitate creative idea generation occurred, but not frequently. Rather, participants frequently reported evaluations of whether concepts should not be selected for concept combination and subsequent idea generation. Despite the presence of predictions, and evaluations in particular, no measurable impact on how the idea generation process was executed was found.

Speculatively, this finding can be interpreted as evidence that predictions and evaluations that form part of concept selection do not necessarily affect how the idea generation process is executed, as measured with fluency, flexibility, and within-concept fluency. This, of course, does not mean that there is no effect on idea generation whatsoever. Previous research, for example, found that selecting concepts that are semantically further apart affect the originality and usefulness of ideas that are generated as a result of conceptual combination (Gibbert et al., 2012). Given previous research, it may be the case that predictions and evaluations that form part of concept selection (and the repetition thereof) have more a qualitative than a quantitative effect (which was assessed in the present study) on idea generation.

Third, the results indicated that the actions conceptual combination and idea generation (and the repetition thereof) are also driven by both predictions and evaluations. That is, predictions about the continued use of the same concepts for idea generation, and evaluations suggesting that the same concepts can facilitate (continued) idea generation, occurred frequently in the participant’s reports about the reasons for their actions taken during the idea generation process. These predictions and evaluations also correlated with how the idea generation process was executed. Participants that reported predictions tended to generate more ideas, and more ideas per concept used. However, these findings were not significant. When participants reported no predictions and no evaluations, they had also used fewer concepts than when they reported only predictions or only evaluations, although the latter was not significant. Moreover, despite a positive correlation between the amount of ideas generated and the amount of concepts used, a rough comparison between their results show that the effects of predictions and evaluations differ (Table 5, model 3), and adding the amount of ideas as a covariate to that same model did not change the results. This suggested that, indeed, these effects were not driven by the amount of ideas that were generated. Complementarily, when participants reported no predictions and no evaluations, they also generated less ideas per concept. Although the former was not significant. In all cases, no differences were found for the amount of ideas generated, the amount of concepts used, and the amount of ideas generated per concept, when comparing reports that contained only predictions or only evaluation, with reports that contained both predictions and evaluations.

Speculatively, these findings can be explained in the following ways. One way to explain the results is that the reports about predictions about the continued and successful use of the same concepts for idea generation and positive evaluations drive a dual process that is commonly hypothesized to characterize the way in which an idea generation process is executed (Nijstad et al., 2010). That is, people tend to either produce more ideas through the persistent use of a few concepts, raising within-concept fluency. This can be explained by the finding that, possibly, participants that reported predictions had also generated more ideas and had generated more ideas per concept used, or people tend to produce more ideas though the use of many different concepts, lowering within-concept fluency. This can be explained by the finding that when participants reported no prediction and no evaluations, less concepts but not less ideas were produced than when they reported only predictions or only evaluations. In these cases, within-concept fluency was also lowered. Another way to explain these results is that, as in our explanation about the results about switching between concept selection and conceptual combination and idea generation, no predictions and no evaluations signal stagnation somewhere in the idea generation process. However, in this case, they may also signal increased switching itself – indicating the production of more ideas through the use of more different concepts. As such, this finding aligns with previous research on dual process models of idea generation (Nijstad et al., 2010). The findings of the present study add that conceptual combination and idea generation (and the repetition thereof) can be driven explicitly by spontaneous predictions about the continued use of the same concepts for idea generation and evaluations suggesting that the same concepts can facilitate (continued) idea generation.

### Limitations

Of course, there are several limitations to the presented study. This is partly due to the exploratory nature of the study and partly due to other more specific methodological choices that were made, some of which we wish to discuss in more detail.

First, the use of three probes to capture predictions and evaluations during the idea generation task affects the validity of the results. It could, for example, be argued that after the first probe was presented, participants were primed to think about predictions and evaluations during idea generation, in preparation for the next probe. This could have helped to capture predictions and evaluations but could also have steered participants into applying predictions and evaluations more frequently, and thereby confounding the results. Moreover, because of using only three probes, and dummy-coding their results into nominal variables, the exact relationships between the predictions and evaluations made could not easily be assessed. Indeed, one would expect that what truly drives idea generation are predictions and evaluations of whether actions taken match or mismatch with these predictions, and is further influenced by their frequency (Dietrich, 2015; Dietrich and Haider, 2015; Yang and Li, 2018). Capturing the relationships between predictions, actions, and evaluations would therefore require a higher resolution than can be achieved with probes, as increasing the number of probes would be at the cost of the ability to actually execute the idea generation process. As such, a different method is needed to achieve this increased resolution.

Second, and related, one limitation was also due to the questions that formed part of the administered probes. That is, the relatively few predictions that were reported about concept selection (and the repetition thereof) could well be an artifact of the way that the present study was set up. It may well be that, counter to our initial conjectures, simply asking participants why they decided not to use certain concepts (Figure 3, question 2) did not capture the phase in which predictions occur during concept selection, but rather only the phase in which evaluations occurred. For example, people apply a range of heuristics during concept selection, such as concept selection based on similarity judgments (Gibbert et al., 2012), use their experiences they gain during idea generation, and domain knowledge. Asking people why they applied a particular heuristic may have yielded better insight into the type of predictions that occurred, whereas asking why they decided to use certain concepts would help capture the subsequent evaluations that would have taken place.

Third, the way the creative task was structured helped participants to report the reasons for their actions taken, which could then be coded into predictions and evaluations. The use of probes, for example, interrupts the idea generation process at random times. However, regular interruption is something that one attempts to prevent during idea generation (Madjar and Shalley, 2008). Moreover, to facilitate insight for both the participants and the researchers into how concept selection, conceptual combination, and idea generation took place, concepts were elicited as part of an association task, and this was done prior to and thus separately from the idea generation task. Although it is common to attempt to understand a problem prior to idea generation, this was made a linear step-wise process in the present study, whereas in real-world creative processes, people are free to move between the thinking processes and actions involved in understanding the problem they are working on and the generation of ideas (Isaksen et al., 2010; Mumford et al., 2012). This freedom to integrate new associations that arise during idea generation into our understanding of a problem, to later help facilitate the emergence of new conceptual combinations and idea generation, may in itself lead to (different kinds of) predictions and evaluations. As a consequence, these predictions and evaluations may not have occurred during the presented study, which affects the ecological validity of the study presented here.

Finally, there are several other limitations that should be mentioned that have implications for interpreting and building further upon the results. First, the study was conducted by using a marketing task only. Although this choice likely improved external validity as the participants were known to have at least some knowledge about marketing, a basic requirement for effective idea generation (Mumford et al., 2012; Abraham, 2018), this also introduced some uncertainty about the degree to which these results can be generalized to idea generation in other domains than marketing. Secondly, due to the presence of the researcher during the study, we cannot rule out any effects on the results due to possible socially desirable behaviors by the participants. Third, differently colored sticky notes were used by the participants to self-report the reasons for the actions taken. However, these were not counterbalanced. In light of recent findings of color effects on idea generation (e.g., Lichtenfeld et al., 2012), it can therefore not be ruled out that these may have affected the results. Fourth, the novelty of the method led to difficulties in statistically justifying the sample size needed for the study (see footnote 1). The choice not to statistically justify the sample size, however, does introduce uncertainty about whether the study is sufficiently powered, subsequently introducing uncertainty about the likelihood that type I and type II errors may have occurred (Cohen, 1992). Fifth, please note that when interpreting the correlations between fluency, flexibility, and within-concept fluency presented in Table 4, and when comparing further results that involve these variables, fluency and flexibility are confounded (Forthmann et al., 2018). This introduces uncertainty about how these results should be interpreted. That said, further testing also suggested that effects of prediction and evaluation on flexibility that were found were unlikely to be driven by fluency.

### Future Work

After taking this first look at the predictions and evaluations that drive the idea generation process, a second and third look seem justified.

Firstly, we propose that future work should investigate the function of predictions and evaluations in the idea generation process in more detail. Previous research outside the domain of creativity suggests that predictions about the consequences of thinking processes and actions serve to reduce uncertainty about whether these actions help achieve a set goal (Hohwy, 2013; Clark, 2015). In other words, these are the mechanisms that help select and learn about what strategies are effective for solving a given problem. One unpublished study within the domain of creativity supports this to some extent, as it showed that that instructing participants to form predictions about an idea in light of its possible future implementation reduced uncertainty (Bruijninckx and de Rooij, unpublished). In line with this, Dietrich and Haider (2015) proposed that how predictions and evaluations form part of the idea generation process reflects different strategies that people undertake to produce creative ideas. The results of the present study already provide preliminary evidence for this. For example, in the present study, people tended to either produce more ideas through the persistent use of a few concepts, raising within-concept fluency, or produce (more) ideas through the inclusion of more different categories, decreasing within-concept fluency, reflecting two different idea generation strategies that appeared to be driven by different combinations of predictions and evaluations (Table 5, bottom), which aligns with Nijstad et al.’s (2010) dual-pathway model of idea generation. Opportunities for future work therefore lie in the further investigation of the function of predictions and evaluations in the idea generation process.

Secondly, and related to this opportunity, we propose that future work should investigate the typology of predictions and evaluations that occur. Although not reported in detail in the present paper, the participants reported a rich variety of reasons for the actions they undertook during idea generation. Intuitively, this suggests that there are different types of predictions and evaluations that commonly form part of the idea generation process. Table 2 already shows this to some extent, where some predictions and evaluations were explicitly about the consequences of using a concept or generating an idea for its implementation, e.g., predictions about whether a concept would facilitate the generation of ideas that will sufficiently reach a target group, and other predictions and evaluations were explicitly about maintaining the idea generation process itself, e.g., predictions about whether a concept is likely to enable the sustained production of ideas. Understanding details of the contents of predictions and evaluations can therefore help to uncover not only how the creative process is executed but also how these shape specific qualities of ideas, e.g., originality and effectiveness. Indeed, previous scholars have also hinted upon the relevance of such a study, suggesting that relatively little is known about what process evaluations are involved in it (Mumford et al., 2012). Opportunities for future work therefore also lie in the further investigation of the different types of predictions and evaluations that underlie the idea generation process.

## Data Availability Statement

The data supporting the conclusions of this manuscript will be made available by the authors, upon request, and without undue reservation, to any qualified researcher.

## Ethics Statement

This study was reviewed and approved by the Research Ethics and Data Management Committee of Tilburg School of Humanities and Digital Sciences, Tilburg University. The participants provided their written informed consent to participate in this study.

## Author Contributions

JV and AR wrote the manuscript and developed the theory and method. JV produced the materials, collected the data, and conducted the qualitative analysis. AR conducted the statistical analysis.

## Conflict of Interest

The authors declare that the research was conducted in the absence of any commercial or financial relationships that could be construed as a potential conflict of interest.

## Footnotes

1 Note that the sample size for this study was not statistically justified. Firstly, this was not done because the novelty of the method used and the topic of the study did not enable us to use data from prior work to do a power analysis (Cohen, 1992). Secondly, as the study also has a substantial qualitative component, sample size also needs to be determined by following conventions of qualitative research, e.g., reaching saturation. Thirdly, heuristics for mixed-method studies exist (Onwuegbuzie and Collins, 2007), but again are difficult to interpret given the novelty of the method used. Therefore, the sample size was a judgment call made by the authors that aimed to collect a dataset that allowed us to take a first quantitative and qualitative look at the occurrence and functioning of predictions and evaluations during idea generation, within the constraints of available time and other resources.
